# Natural progression of the pubofemoral distance with age and its correlation with future acetabular index

**DOI:** 10.1007/s00330-023-09579-z

**Published:** 2023-04-04

**Authors:** Wen-Chieh Chang, Kuei-Hsiang Hsu, Yu-Ping Su

**Affiliations:** 1 Department of Orthopaedics and Traumatology, Taipei Veterans General Hospital, Taipei, Taiwan; 2 Department of Orthopaedics, School of Medicine, National Yang Ming Chiao Tung University, Taipei, Taiwan

**Keywords:** Developmental dysplasia of the hip, Ultrasonography, Radiology

## Abstract

**Objectives:**

This study investigated the progression of pubofemoral distance (PFD) with age and assessed the correlation between PFD and late acetabular index (AI) measurements.

**Methods:**

This prospective observational study was conducted between January 2017 and December 2021. We enrolled 223 newborns who underwent the first, second, and third hip ultrasounds, and pelvis radiograph at a mean age of 18.6 days, 3.1 months, 5.2 months, and 6.8 months, respectively. The difference between PFD measured at serial ultrasounds and the correlation with AI were analyzed.

**Results:**

The PFD increased significantly (*p* < 0.001) at serial measurements. The mean PFD at the first, second, and third ultrasounds were 3.3 (2.0–5.7), 4.3 (2.9–7.2), and 5.1 (3.3–8.0) mm, respectively. The PFD at three ultrasounds were all significantly (*p* < 0.001) and positively correlated with AI, with the Pearson correlation coefficients being 0.658, 0.696, and 0.753 for the first, second, and third ultrasounds, respectively. Using AI as reference, the diagnostic ability of PFD was calculated by the areas under the receiver operating characteristic curve, which was 0.845, 0.902, and 0.938 for the first, second, and third PFD, respectively. For the first, second, and third ultrasounds, PFD cutoff values of ≥ 3.9, ≥ 5.0, and ≥ 5.7 mm, respectively, yielded the greatest sensitivity and specificity in predicting late abnormal AI.

**Conclusion:**

The PFD naturally progresses with age and is positively correlated with AI. The PFD has potential for predicting residual dysplasia. However, the cutoff for abnormal PFD values may require adjustment according to the patient’s age.

**Key Points:**

*• The pubofemoral distance measured in hip ultrasonography naturally increases as the infant’s hips mature*.

• *The early pubofemoral distance demonstrates a positive correlation with late acetabular index measurements*.

• *The pubofemoral distance may help physicians predict abnormal acetabular index. However, the cutoff for abnormal pubofemoral distance values may require adjustment according to patient’s age*.

## Introduction

Developmental dysplasia of the hip (DDH) is an abnormal development of the osseous and soft tissue of the hip joint, leading to insufficient coverage of the femoral head. DDH has varying clinical manifestations and typically presents as mild dysplasia, subluxation, or frank dislocation. The reported incidence of DDH is 3–5 per 1000 newborns [1, 2]. The standard screening strategy for DDH in infants before the age of 4 months involves universal physical examination for hip stability followed by selective hip ultrasonography for newborns with either abnormal physical examination findings or risk factors [2, 3]. The Graf method and the femoral head coverage (FHC) ratio are the most extensively used ultrasonography parameters for DDH screening; both tools provide quantitative results and accurate diagnoses [4–6]. After age of 4 months, femoral head ossification starts and the acetabular index (AI) measured from anteroposterior pelvic radiography can be used to evaluate acetabular morphology.

Couture et al and Tréguier et al have recently developed a simple ultrasound method based on the pubofemoral distance (PFD), which may be adopted as an adjunctive tool for DDH screening [7, 8]. The PFD can be an accessional ultrasound parameter in addition to the Graf method and FHC ratio to serve as a fast, low-cost, highly sensitive, and specific screening tool [7–11]. Moreover, the PFD can be used to assist Graf method and FHC ratio to evaluate the effectiveness of hip reduction and to monitor patients underwent Pavlik harness treatment with high intra- and inter-observer reliability [12, 13].

Early ultrasound findings may predict late AI measurements. Studies have extensively explored the correlation between ultrasound and AI measurements for DDH screening; nevertheless, most of such studies have focused on ultrasound screening performed by the Graf method and the FHC ratio [14–20]. Evidence regarding the correlation between the PFD and AI is relatively scarce. Whether an increase in PFD is associated with an increase in AI is uncertain. In addition, similar to the alpha angle in the Graf method and the FHC ratio, the PFD may differ with age. Although studies using the Graf method and the FHC ratio for screening have adequately documented spontaneous improvements in hip morphology, the natural history of the PFD is less extensively discussed and remains unclear [21–24].

Accordingly, the primary objective of this study was to investigate the progression of the PFD with age in consecutive infants. The secondary objective was to assess the correlation between the PFD and late AI measurements.

## Materials and methods

This prospective observational study was conducted from January 1, 2017, to December 31, 2021. We included all newborns who were delivered at our institution or were referred to our hospital for DDH. Newborns with neurogenic dislocation, syndromic dislocation, or a history of Pavlik harness treatment were excluded. The study was performed in accordance with the ethical principles set out in the 1964 Declaration of Helsinki and was approved by the institutional review board at our institution (IRB number 2017–06-010AC).

A neonatologist performed the Barlow and Ortolani maneuvers on all newborns to screen for hip stability [25, 26]. Hip status was categorized according to the Barlow and Ortolani test results into three groups: stable hips (involving a fixed position of the hip center during examination), subluxatable hips (involving any laxity of the hip center without dislocation), and dislocatable or dislocated hips (involving complete displacement of the hip center from the acetabulum). Hips that were considered to be subluxatable required confirmation from two senior pediatric orthopedic surgeons. In addition to physical examination findings, risk factors such as the female sex, twin pregnancy, firstborn birth order, breech presentation, or a family history of DDH were documented [2].

**Fig. 1:**
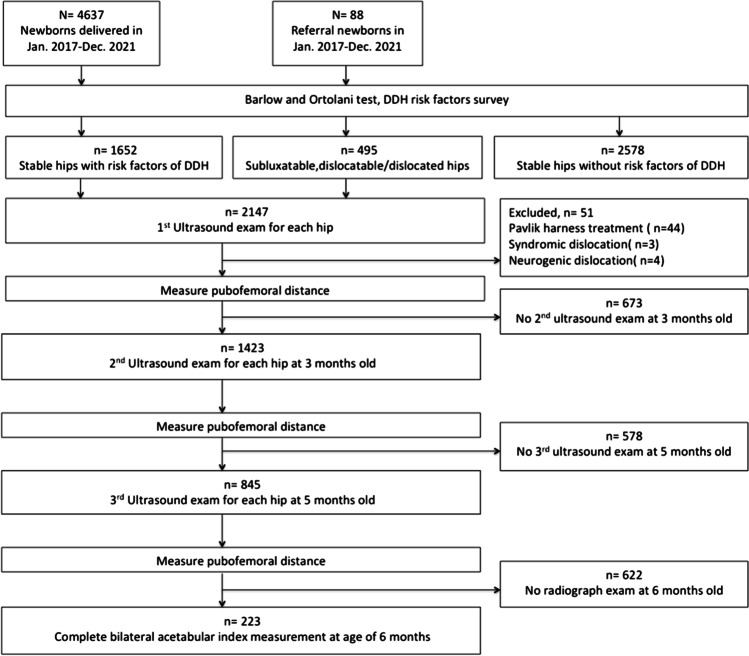
Flowchart of patients’ inclusion and exclusion in this study. DDH, developmental dysplasia of the hip

All newborns were divided into three groups according to their Barlow and Ortolani test results and risk factor status: group 1, comprising newborns who had stable hips but had DDH risk factors; group 2, comprising newborns with subluxatable, dislocatable, or dislocated hips regardless of the presence of risk factors; and group 3, comprising newborns with stable hips and no risk factors (Fig. 1).

After informed consent was obtained from the parents, the newborns in groups 1 and 2 were subjected to serial ultrasound examinations of the bilateral hips. The first ultrasound examination was performed immediately after the hip physical examination and risk factor survey in the newborn nursery. The second and third ultrasound examinations, conducted at the outpatient clinic, monitored hip morphology at the age of approximately 3 and 5 months.

A pediatric orthopedic surgeon certified in musculoskeletal ultrasound examination performed the hip sonography using a 7.5-MHz linear transducer (LOGIQ e ultrasound, GE Healthcare) positioned perpendicular to the hip joint, with no tilting during the examination. Each of the patients was placed in the lateral decubitus position, with the hips slightly flexed, adducted, and internally rotated throughout the ultrasound examination [27]. The criteria for the selection of qualified hip ultrasound images were as follows: inclusion of the lower iliac margin at the triradiate cartilage, the labrum, the deepest point of the acetabulum, the pubis, and the chondroosseous border of the proximal femur [27]. For each indicated participant, the most representative image out of three repeated image acquisitions at the bilateral hips was selected for ultrasound interpretation.

**Fig. 2:**
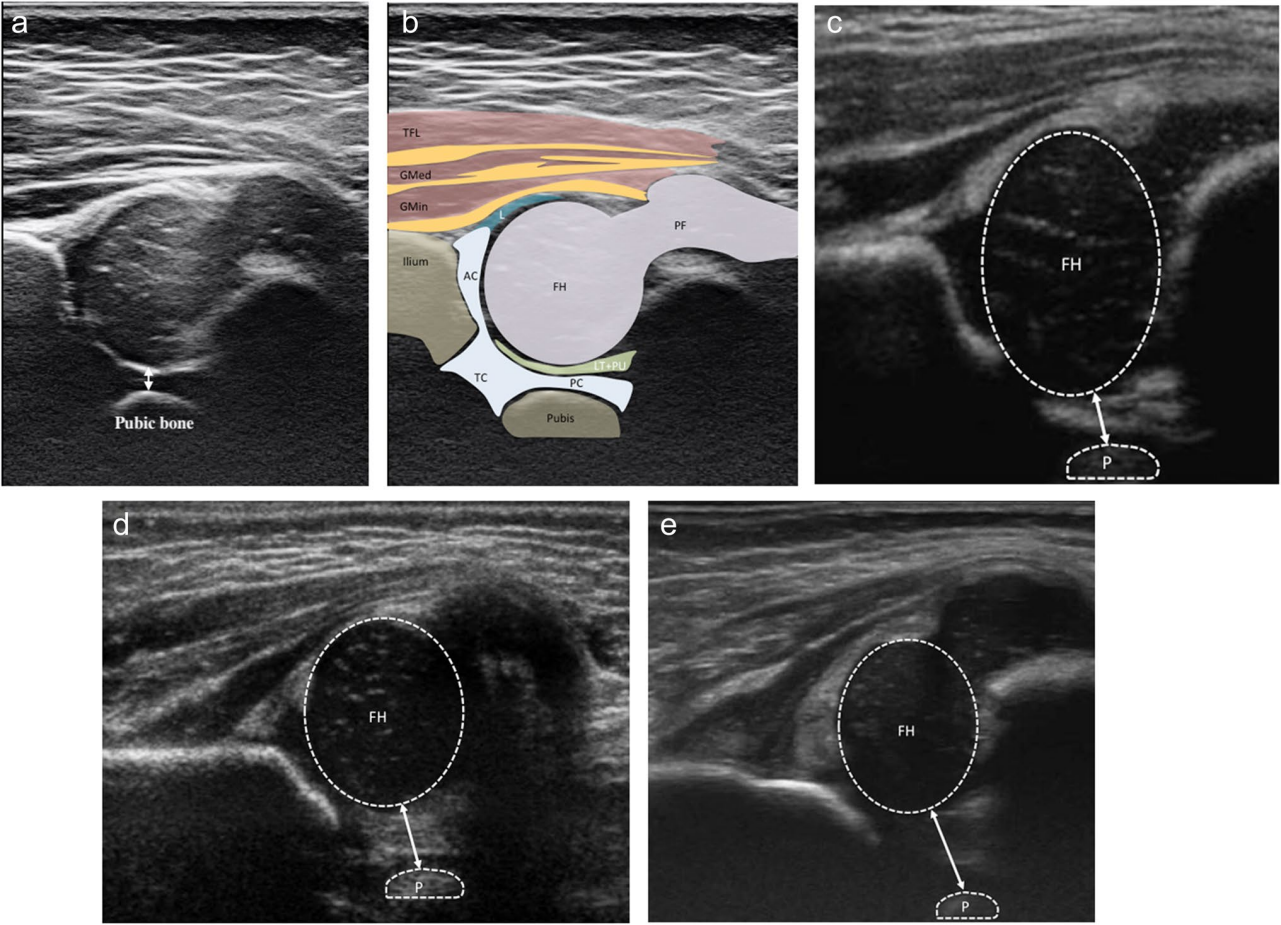
**a** A 1-month-old female infant with normal Graf I left hip. The PFD (double-headed arrow) is measured as the shortest distance between the pubic bone and the medial margin of the femoral epiphysis, which is 2.6 mm in this infant. **b** The drawing of main anatomic landmarks and the pelvic structure identified for PFD measurement in **a**. **c** A 5-week-old male infant with Graf IIa left hip. The hip is physiological immature and the hip morphology gradually returned to normal at follow-up. The PFD (double-headed arrow) is 4 mm for this infant. **d** A 3-week-old female infant with Graf IIc left hip, the femoral head coverage is only 35%, and the hip joint is subluxatable during Barlow maneuver. The PFD (double-headed arrow) increased to 6.8 mm in this patient. **e** A 1-month-old female infant with dysplastic left hip and femoral head dislocation, leading to enlarged PFD (double-headed arrow). The PFD increased to 8.8 mm in this case. PFD, pubofemoral difference; TFL, tensor fasciae latae; GMed, gluteus medius; GMin, gluteus minimus; TC, triradiate cartilage; PC, pubic cartilage; AC, acetabular cartilage; FH, femoral head; PF, proximal femur; L, labrum; LT, ligamentum teres; PU, fatty pulvinar; P, pubis

The PFD was measured as the shortest distance between the pubic bone and the medial margin of the femoral epiphysis (Fig. 2) [7, 8]. Couture and Tréguier performed their ultrasound examination in the supine position when they introduced the PFD; nevertheless, subsequent studies have demonstrated that the PFD measured in the lateral decubitus position can also yield high sensitivity and specificity in detecting hip instability [10]. At our institution, ultrasound examinations are performed in the lateral decubitus position in accordance with Graf’s guidelines for hip sonogram positioning [27].

**Fig. 3:**
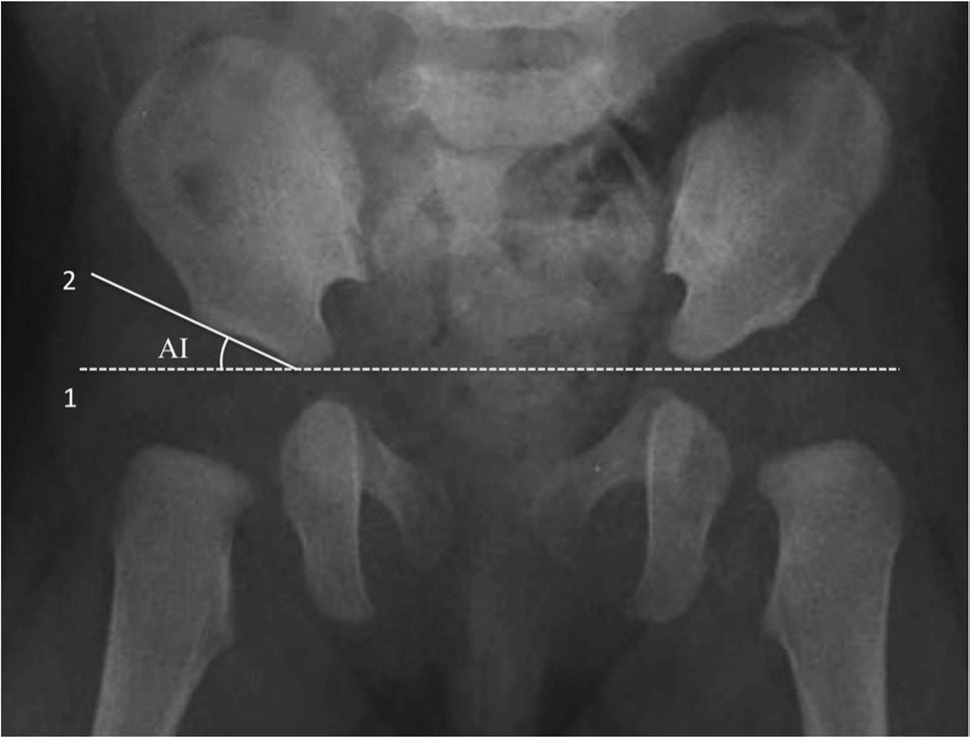
The acetabular index (AI) is the angle formed by Hilgenreiner line (Line 1) and a line drawn from the triradiate epiphysis to the lateral edge of the acetabulum (Line 2)

An anteroposterior plain film radiograph of the pelvis was conducted for each of the patients at the age of 6 months in order to monitor their hip development. The AI is defined as the angle between the Hilgenreiner line and a line drawn from the triradiate epiphysis to the lateral edge of the acetabulum (Fig. 3). Although most current studies establish the normal AI range based on various ages, it should be noted that there is evidence that suggests the female may have higher AI than male before age of 7 years [28]. The mean AI for females is approximately 3° higher than for males when measured at age of 6 months to 1 year [28]. Based on the definition for normal AI range from most literatures, we used the mean AI for infants at the same age to determine abnormal AI regardless of patient’s sex in this study. An AI value exceeding 23.5° at 6 months of age is considered abnormal [29].

A senior pediatric orthopedic surgeon measured all PFD values in this study. A pediatric orthopedic fellow who was blinded to the ultrasound findings measured all AI values. To ensure consistency of measurements for the two observers, one observer measured the PFD for anonymized sonograms captured from 50 hips, and the other observer measured the AI for anonymized radiograph from the same image set. The same observers, blinded to the patients’ information and their previous results, repeated the image interpretation for the same 50 hips 3 weeks later. The results showed high consistency between two measurements for two observers, with the intraclass correlation coefficient being 0.95 (95% confidence interval [CI]: 0.91–0.99) for the PFD and 0.93 (95% CI: 0.90–0.96) for the AI measurement.

The images from patients who completed three ultrasound examinations and the pelvic plain films captured at 6 months were analyzed. The primary outcome measure was the difference between the PFD values measured from three hip ultrasound images. The secondary outcome measure was the correlation of the PFD values measured from three hip ultrasounds with the AI measured at the age of 6 months, and the cutoff for abnormal PFD values using AI as the reference standard.

### Statistical analysis

A paired-sample *t*-test was used to compare the PFD values measured on three consecutive hip ultrasound images. A two-sample *t*-test was used to compare the PFD values between the male and the female groups. The Pearson correlation coefficient was calculated to determine the correlation between the PFD and AI measurements. A receiver operating characteristic (ROC) curve analysis was performed to investigate the diagnostic ability and cutoff for abnormal PFD values, with the AI serving as the reference standard. Statistical significance was defined as *p* < 0.05. All statistical analyses were performed using SPSS (version 22; IBM Corp.).

## Results

From January 2017 to December 2021, a total of 4637 newborns delivered at our institution and 88 infants referred to our institution underwent Barlow and Ortolani tests and a risk factor survey for DDH screening. A total of 1652 newborns who had stable hips but had risk factors and 495 newborns with abnormal physical examination findings underwent the first ultrasound examination for the bilateral hips (Fig. 1). We excluded 51 patients for the following reasons: receiving Pavlik harness treatment (*n* = 44), having syndromic dislocation (*n* = 3), and having neurogenic dislocation (*n* = 4). Moreover, 1423 and 845 patients subsequently underwent the second and third hip ultrasound examinations, respectively, and 223 patients finally underwent anteroposterior pelvic radiography at the age of 6 months (Fig. 1). A total of 223 patients completed three serial hip ultrasounds and the pelvis plain film were included in our final analysis. The mean ages at the first, second, and third ultrasound examinations, and pelvis plain film were 18.6 (0–22) days, 3.1 (2.5–4.2) months, 5.2 (4.2–6.1) months, and 6.8 (5.8–7.6) months, respectively. The characteristics of enrolled patients are summarized in Table 1.

**Table 1:** Patient characteristics

Characteristics, *n* = 223	
Age at 1^st^ hip ultrasound (days)	18.6 (0–22)
Age at 2^nd^ hip ultrasound (months)	3.1 (2.5–4.2)
Age at 3^rd^ hip ultrasound (months)	5.2 (4.2–6.1)
Age at pelvis plain film (months)	6.8 (5.8–7.6)
Female	128 (57%)
Gestational age (weeks)	38.1 (26–41)
Twins	7 (3%)
Birth weight (grams)	2922 (1950–4218)
Firstborn	92 (41%)
Breech presentation	13 (6%)
Family history of DDH	62 (28%)

*DDH* developmental dysplasia of the hip

**Fig. 4:**
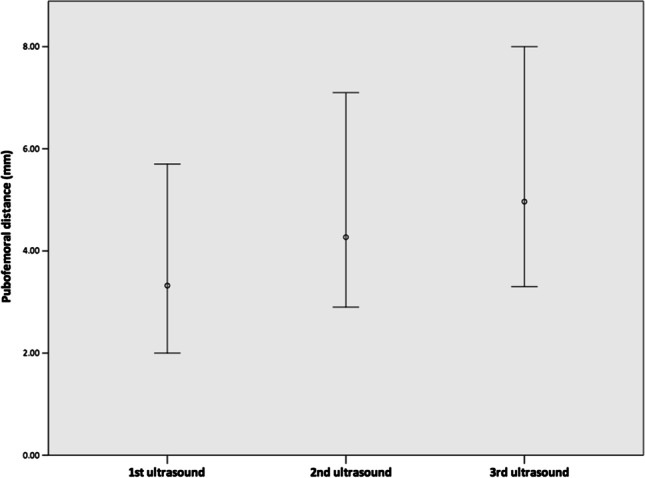
Distribution of the pubofemoral distance measured at three serial hip ultrasounds

Regarding the PFD measured on three serial hip ultrasounds, the mean PFD increased significantly (*p* < 0.001) between each measurement (Fig. 4). The mean PFD of the first, second, and third ultrasounds were 3.3 (2.0–5.7), 4.3 (2.9–7.2), and 5.1 (3.3–8.0) mm, respectively (Table 2). The trend of naturally increased PFD was observed in both boys and girls; however, the mean PFD were not significantly different between sex in three serial ultrasounds (3.1 vs. 3.4 mm, *p* = 0.31; 4.3 vs. 4.2 mm, *p* = 0.47; 5.0 vs. 5.2 mm, *p* = 0.48, Table 2).

**Table 2:** Distribution of pubofemoral distance according to age and sex (in mm)

Age	All *n* = 223			Male *n* = 95			Female *n* = 128		
	Mean	SD	Cutoff (Sen/Spe)^*^	Mean	SD	Cutoff (Sen/Spe)*	Mean	SD	Cutoff (Sen/Spe)*
18.6 (0–22) days	3.3	0.6	3.9 (72.5/90.6%)	3.1	0.6	3.8 (68.2/87.5%)	3.4	0.6	4.0 (69.0/93.4%)
3.1 (2.5–4.2) months	4.3	0.8	5.0 (84.3/91.6%)	4.3	0.8	4.9 (81.8/88.1%)	4.2	0.7	5.0 (89.7/94.7%)
5.2 (4.2–6.1) months	5.1	1.0	5.7 (92.2/94.2%)	5.0	1.0	5.6 (86.4/90.5%)	5.2	0.9	5.9 (96.6/95.2%)

*SD* standard deviation; *Sen* sensitivity; *Spe* specificity

^*^Cutoff value for detecting abnormal acetabular index at age of 6 months

**Fig. 5:**
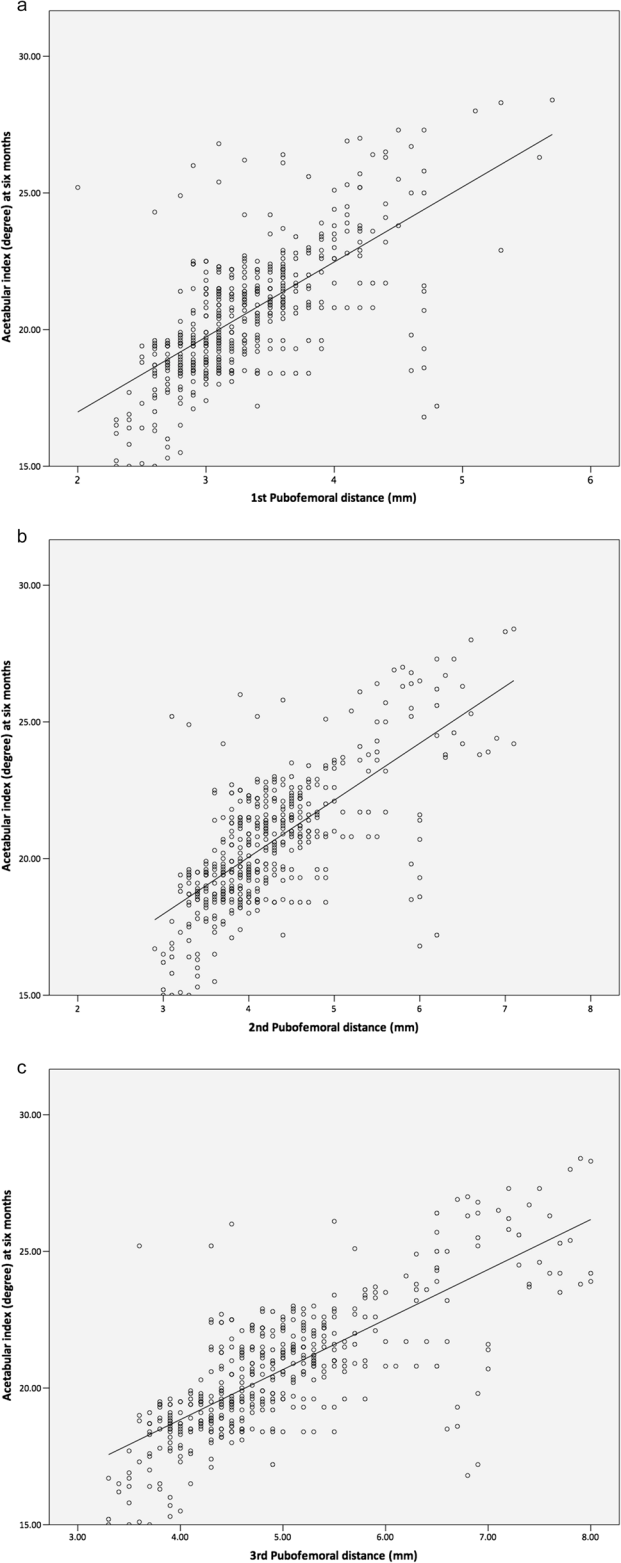
Correlation between the first (**a**), second (**b**), and third (**c**) pubofemoral distances with acetabular index at 6 months

The PFD values measured on three consecutive hip ultrasounds all exhibited a significant positive correlation with the AI measured at the age of 6 months. The Pearson correlation coefficients for the PFD measured on the first, second, and third hip ultrasounds were 0.658 (*p* < 0.001), 0.696 (*p* < 0.001), and 0.753 (*p* < 0.001), respectively. The correlation between the measured PFD values and AI is illustrated in Fig. 5.

**Fig. 6:**
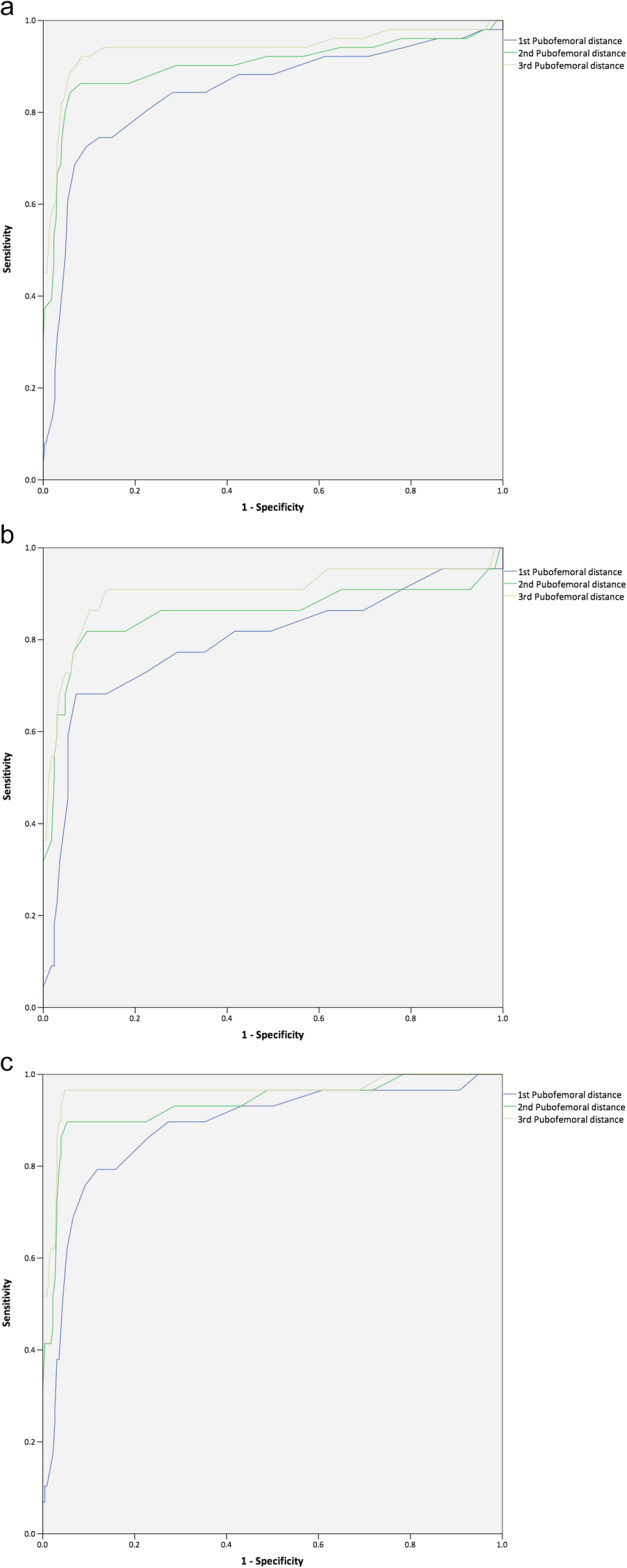
Receiver operating characteristic curve analysis for three pubofemoral distance measurements in all participants (**a**), and male (**b**) and female (**c**) infants using the acetabular index as reference

Using the AI at the age of 6 months as the reference standard, the diagnostic ability of the PFD was assessed by using ROC curve analysis (Fig. 6a). The PFD values from three ultrasound exams all exhibited high diagnostic performance in predicting AI. The areas under the ROC curve (AUC) for the PFD measured on the first, second, and third hip ultrasounds were 0.845 (95% CI: 0.775–0.915), 0.902 (95% CI: 0.839–0.965), and 0.938 (95% CI: 0.888–0.988), respectively.

The ROC curve analysis was further performed for boys and girls (Fig. 6b, c). The PFD value from three hip ultrasounds demonstrated high diagnostic ability to predict abnormal AI in both genders. For the male, the AUC for the PFD measured on the first, second, and third ultrasounds were 0.795 (95% CI: 0.670–0.920), 0.857 (95% CI: 0.734–0.979), and 0.905 (95% CI: 0.808–1.0), respectively. For the female, the AUC for the PFD measured on the first, second, and third ultrasounds were 0.883 (95% CI: 0.808–0.957), 0.935 (95% CI: 0.875–0.994), and 0.963 (95% CI: 0.915–1.0), respectively.

The cutoff value of PFD to exhibit greatest sensitivity and specificity to detect abnormal AI measurement based on age and sex distribution is listed in Table 2.

## Discussion

In this study, the PFD significantly increased with age in newborns. PFD values measured on three serial hip ultrasounds all exhibited a significant positive correlation with AI measured at the age of 6 months, and the early PFD value may be served as an effective tool for predicting late abnormal AI measurements.

Studies have extensively reported that ultrasound parameters used for DDH screening improved with infant hip maturation [21, 22, 24]. However, most of such studies have focused on changes in ultrasound features used in the Graf method and FHC ratio. Although most studies have demonstrated that the alpha angle and FHC ratio increased naturally with age, few studies have considered the natural history of the PFD. Ban et al examined 240 infants aged 0–12 months who had Graf type Ia or Ib hips. They reported that the PFD increased linearly with age [23]. However, their study design involved performing ultrasound examinations for different infants with various ages. Their results suggested that older infants may have higher PFD values, but they did not reveal the natural progression of the PFD or the trend of the PFD with age among the infants.

On the basis of our results, we suggest that PFD values could be affected by the age at ultrasound acquisition. Because the PFD may naturally increase with age, PFD values observed at different ages may account for the various cutoff values of abnormal PFD measurements in previous studies. Tréguier et al defined the cutoff for abnormal PFD values to be 6 mm from patients with varying ages, ranging from < 28 to > 90 days [8]. Teixeria et al proposed that the cutoff for abnormal PFD values should be 4.9 mm when measured in hip flexion, 4.6 mm when measured in a neutral position, or asymmetry > 1.5 mm to contralateral hip [11]. Husum et al suggested that the cutoff for abnormal PFD values should be > 4.4 mm when measured in the lateral decubitus position [10]. Both studies conducted by Teixeria et al and Husum et al have established thresholds for abnormal PFD values in infants aged approximately 1 month. Motta et al reported a mean PFD of 3.5 mm for dysplastic hips in infants aged from 3 days to 6 months [9]. The heterogeneity of patient age in the aforementioned studies may explain the inconsistency for the cutoff values of abnormal PFD. Moreover, whether the patient’s gender can affect PFD interpretation is unknown from current literatures. From our results, we did not observe statistical difference of PFD between sex from three serial ultrasounds. However, the cutoff value for abnormal PFD was slightly different between boys and girls in the ultrasounds performed at various age. The PFD distribution in different gender may require more evidence by research with larger sample scale and more long-term follow-up.

Studies on the correlation between PFD and AI measurements are limited in the previous research. As demonstrated in the literature, the alpha angle and FHC ratio are negatively correlated with AI measurements; that is, patients with a lower alpha angle or FHC ratio are more likely to have a higher AI [14–20]. Nonetheless, previous studies have not clarified whether the same correlation can be observed between PFD and AI measurements. Previous research has defined abnormal PFD values by using the Graf method as the reference standard [8–11]; hence, evidence regarding the association between PFD and AI measurements is extremely limited in the literature. According to our review of current evidence, no large-scale study or quantitative evidence confirming the correlation between PFD and AI measurements was available prior to the present study.

The main strength of this study is that it provides information about the natural progression of the PFD and its correlation with late AI measurements in consecutive infants. The PFD is a relatively novel ultrasound parameter for DDH screening compared with the Graf method and the FHC ratio. Hence, according to our review of the literature, our study may be the first to further discover the natural history of the PFD and its association with radiographic findings. Our results suggest that normal PFD values may vary depending on the timing of observation and that the PFD has the potential to predict residual dysplasia. Further research with a larger sample size and longer follow-up period can more clearly identify the precise cutoff for abnormal PFD values at various ages.

Our study has several limitations. First, this is a study with a relatively small sample size and a short follow-up period. Second, lack of routine dynamic ultrasound examinations for DDH screening may have affected the PFD interpretation. Finally, we did not calculate the thickness of the pulvinar or pubic cartilage separately when measuring the PFD; one study suggested that both factors should be considered when interpreting PFD results [30].

In conclusion, the PFD naturally progresses with age. The PFD not only demonstrates a significant positive correlation with AI measurements but also has the potential for predicting residual dysplasia. The patient’s age may need to be considered when determining the cutoff for abnormal PFD values. In conjunction with Graf method and FHC ratio, the PFD measurement can help current ultrasound method for DDH screening.

## Abbreviations

AI: Acetabular index
DDH: Developmental dysplasia of the hip
FHC: Femoral head coverage
PFD: Pubofemoral distance

## Declarations

### Guarantor

The scientific guarantor of this publication is Yu-Ping Su, MD, PhD, the corresponding author of this study.

### Statistics and biometry

The authors acknowledge the statistical support of the Research Center of Biostatistics, Taipei Veterans General Hospital.

### Methodology

• Prospective

• Observational

• Performed at one institution
